# Optical Activity of Metal Nanoclusters Deposited on Regular and Doped Oxide Supports from First-Principles Simulations

**DOI:** 10.3390/molecules26226961

**Published:** 2021-11-18

**Authors:** Luca Sementa, Mauro Stener, Alessandro Fortunelli

**Affiliations:** 1 CNR-IPCF, Consiglio Nazionale delle Ricerche, 56124 Pisa, Italy; 2 Dipartimento di Scienze Chimiche e Farmaceutiche, Università di Trieste, 34127 Trieste, Italy; 3 CNR-ICCOM, Consiglio Nazionale delle Ricerche, 56124 Pisa, Italy

**Keywords:** metal clusters, optical photo-absorption, Time-Dependent Density-Functional Theory (TDDFT), cluster/oxide interface, first-principles modeling, photo-enhanced processes

## Abstract

We report a computational study and analysis of the optical absorption processes of Ag_20_ and Au_20_ clusters deposited on the magnesium oxide (100) facet, both regular and including point defects. Ag_20_ and Au_20_ are taken as models of metal nanoparticles and their plasmonic response, MgO as a model of a simple oxide support. We consider oxide defects both on the oxygen anion framework (i.e., a neutral oxygen vacancy) and in the magnesium cation framework (i.e., replacing Mg^++^ with a transition metal: Cu^++^ or Co^++^). We relax the clusters’ geometries via Density-Functional Theory (DFT) and calculate the photo-absorption spectra via Time-Dependent DFT (TDDFT) simulations on the relaxed geometries. We find that the substrate/cluster interaction induces a broadening and a red-shift of the excited states of the clusters, phenomena that are enhanced by the presence of an oxygen vacancy and its localized excitations. The presence of a transition-metal dopant does not qualitatively affect the spectral profile. However, when it lies next to an oxygen vacancy for Ag_20_, it can strongly enhance the component of the cluster excitations perpendicular to the surface, thus favoring charge injection.

## 1. Introduction

In the search of devices able to efficiently capture solar energy and make full use of its potential within a renewable energy cycle [1], light-absorber oxide semi-conductors promoted by deposited metal nanoparticles represent an important class of materials, in both solar-cell [2] and photocatalytic [3,4,5,6,7,8,9,10] technological applications. Noble metal particles in fact exhibit Localized Surface Plasmon Resonances (LSPR), i.e., intense absorption peaks in the optical region of the solar spectrum due to collective oscillations of their valence electrons [11]. These LSPR optical excitations can capture light and then transfer its energy as an excitation into the valence band of the semi-conductor. This effect can be exploited in solar cells [8,12]. This effect can be also used to promote photo-chemical reactions and further enhanced by combining it with materials nano-structuring [5,13]. Although these phenomena are supported by sound evidence in experiments, their precise theoretical foundation and detailed modeling are less well-defined. This is due to the difficulties involved in achieving accurate simulations of the optical excitations of large and composite systems, in which nanoparticle/substrate interface effects play an important role, similar to the important role played by interface effects in the analogous field of dye-promoted solar cells [14]. It can be argued that it is necessary to employ an atomistic and quantum-mechanical level of theory in order to properly describe these interface effects [15]. However, in previous work, the metal particle and the light-absorbing semi-conductor have often been treated as separate entities, altogether neglecting particle/substrate interfacial effects. This is technically convenient because it simplifies the problem by bringing it back to well-studied separate LSPR and solid-state excitation phenomena [16,17,18], but this is obtained at the cost of missing potentially important effects. Recently, progress has been made, and the optical spectra of small sub-nanometer clusters on reducible oxides have been simulated using an explicit atomistic QM description (e.g., Ag_5_ on TiO_2_ [19]), leading to very interesting results. However, due to finite-size effects at their extreme [20], the effects observed in sub-nanometer clusters were quantitatively far from the substantial enhancement in light-capture efficiency found in some experiment and required in device applications.

To provide rigorous information on this appealing topic, here we conduct a series of time-dependent density-functional theory (TDDFT) simulations on Ag_20_ and Au_20_ metal clusters supported on the (100) surface of a simple oxide, MgO(100). For the support, we consider both the regular facet of MgO(100) and systems including point defects. In particular, we consider defects both on the oxygen anion framework (i.e., a neutral oxygen vacancy, that is, a common color center or “Farbe-center” or F-centre in bulk and surface oxide systems [21]), and in the magnesium cation framework (specifically, replacing Mg^++^ with selected transition metals: Cu^++^ or Co^++^). The Ag_20_ and Au_20_ metal clusters are small enough to be amenable to Quantum-Mechanical (QM) TDDFT simulations and have widely been employed as models of plasmonic metal particles [22,23,24], although their main absorption peak lies in the near-UV rather than in the optical region of the spectrum. MgO is a wide-band-gap insulator rather than a narrow-band-gap semi-conductor, but furnishes a practical model to investigate the influence of a charge-separated oxide support on the absorption spectra and the excitations of supported metal clusters, thus allowing one to study effects such as the shape of the induced response and its relationships with the oxide surface. Moreover, the choice of the MgO support allows us to study plausible point defects, such as neutral oxygen vacancies or substitutional dopants, and explore their effects on the collective excited states of the composite system. In the future, the information resulting from the present study could be exploited to couple the excitations we describe hereafter with the excitations in the conduction band of a semi-conducting reducible oxide support. In this way, one will achieve that disentanglement of the cluster/substrate composite systems that is needed for simplified understanding and predictions, but in a way that is more controlled and atomistic-QM-based than assuming that the cluster excitations are identical to those of the clusters in the gas-phase as often done in previous work.

As major results, we find a broadening (fragmentation) and a red-shift of the Ag_20_ and Au_20_ metal cluster intense excitations. For example, already the least perturbing support—the MgO(100) regular surface without defects—induces a significant splitting (by ≈ 0.6 eV) of the main absorption pre-plasmonic peak of Ag_20_ into two peaks: (i) a red-shifted lower-energy component, with its transition dipole moment essentially parallel to the oxide surface, and (ii) a higher-energy component, only slightly red-shifted, with its transition dipole moment essentially perpendicular to the oxide surface. The presence of an oxygen vacancy as a defect in the support further strongly enhances these phenomena due to the mixing of the excited states localized on the cluster and on the defect. An even more extensive mixing for Ag_20_ is finally achieved when a transition-metal dopant is present as a substitutional defect in the support next (first-neighbor) to an oxygen vacancy. In Ag_20_, the co-presence of a transition metal dopant and an oxygen vacancy affects the orientation of the transition moment dipole of the cluster excitations with respect to the surface, an effect that is of potential interest in charge-injection or energy-transfer phenomena.

## 2. Results and Discussion

In the following, we will first present results and analysis on the fragments that will enter into our cluster/support composite systems. This will provide both reference data against which to assess the effect of cluster adsorption on the spectra and a validation of our results by comparison with previous simulations or experimental data. Then, we will go on to present and discuss results on the cluster-deposited systems.

### 2.1. Optical Response of the Fragment Systems

We performed TDDFT simulations on the geometries of the following fragments:

- Ag_20,_
- Au_20,_
- MgO(100)-reg, i.e., the non-defected MgO(100) surface,
- MgO(100)-Ovac, i.e., MgO(100) exhibiting an oxygen vacancy at the surface,
- MgO(100)-Cu, i.e., MgO(100) with one Mg^2+^ cation replaced by one Cu^2+^ cation,
- MgO(100)-Ovac+Cu, i.e., MgO(100) with an O-vacancy next to a Cu^2+^ substitution,

The geometries of these systems are illustrated in Figures S1 and S2 of the Supplementary Materials (SM), as well as reported as Cartesian coordinates in the SM (in particular, the finite-cluster model for the oxide is pictorially illustrated in Figure S1 of the SM, see the discussion in the Materials and Methods).

**Figure 1:**
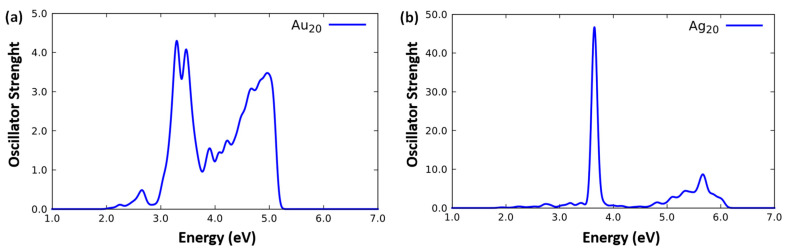
Simulated TDDFT/PBE0 photo-absorption spectrum of: (**a**) Au_20_ and (**b**) Ag_20_ simulated at the TDDFT level on their relaxed geometries using the ORCA code.

The optical absorption spectra of Ag_20_ and Au_20_ derived using the ORCA code (ADF and NWChem gave very similar results) are presented in Figure 1. Both clusters exhibit intense absorption peaks in the near-UV region [24,25]. It can be recalled that the absorption peak of Ag_20_ is more intense (as can be seen from the higher values of the oscillator strengths) and possess a more plasmonic-like character (as can be seen from its narrower and single-peak shape), whereas that of Au_20_ is somewhat more molecular-like due to the coupling between *s*- and *d*-band excitations, as discussed in previous literature [24,25]. However, it can be noted that the two lowest-energy peaks of Au_20_ are at ≈ 3.1 and ≈ 3.3 eV, respectively, thus closer to the optical region than the very intense single peak of Ag_20_ around ≈ 3.7 eV. For comparison, we have also simulated the spectra of Ag_20_ in the geometry extracted from the Ag_20_/MgO(100)-reg interacting system: the corresponding spectra are reported in Figure S3 of the SM and will be mentioned in the next subsection.

**Figure 2:**
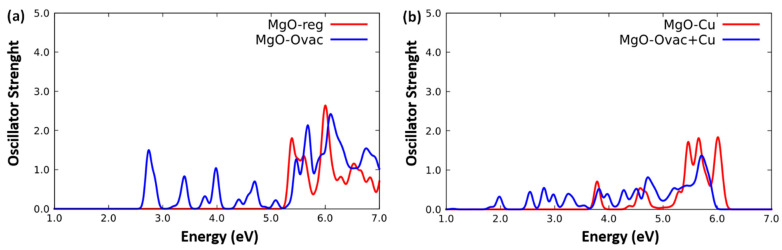
Simulated TDDFT/PBE0 photo-absorption spectra of: (**a**) MgO(100)-reg and MgO(100)-Ovac, and (**b**) MgO(100)-Cu, and MgO(100)-Ovac+Cu using the ORCA code.

The spectra of the systems: (a) MgO(100)-reg, MgO(100)-Ovac, and (b) MgO(100)-Cu, MgO(100)-Ovac+Cu, obtained using the ORCA code are reported in Figure 2a,b. Starting from regular MgO(100), we find an absorption threshold at around 5.6 eV that compares reasonably well with the experimentally observed surface band gap at around 6.0–6.3 eV [26]. Note that ADF predicts a higher threshold at around 5.9 eV, due to its slightly smaller basis set and the use of frozen-core atoms rather than pseudopotentials at the QM/MM boundary. In the ADF simulation, the HOMO of MgO(100)-reg has an orbital energy at around −6.9 eV and the LUMO has an orbital energy at around −0.7 eV, thus a band-structure gap of 6.2 eV, that is reduced to 5.9 eV by couplings within the TDDFT Casida matrix. The importance of these couplings is underlined by the fact that the Hybrid Diagonal Approximation [27], also implemented within ADF, underestimates the absorption threshold to 5.6 eV. These results are consistent with a band gap of 5.9 eV we previously predicted on a smaller MgO finite cluster using the NWChem code [20]. In addition to the regular-MgO absorption threshold at ≈ 5.6 eV, the MgO(100)-Ovac system exhibits a set of lower-energy excitations associated with the oxygen-vacancy defect, whose lowest-energy peak is predicted by ORCA at ≈ 2.8 eV, while ADF puts it at higher energy at 3.1 eV. These values are reasonable: experimentally, the neutral oxygen vacancy has an absorption peak at around 5 eV, in agreement with accurate post-DFT simulations [28], but this excitation energy value is expected to be substantially reduced at the surface due to the reduction in confinement effects at the surface and the consequently increased polarizability of the electron pair localized in the vacancy [21]. Considering now the effect of a cation replacement, we found that the presence of a Cu substitution in MgO(100)-Cu and MgO(100)-Ovac+Cu does not qualitatively change the spectra. However, the presence of an electron hole in the *d*-band of Cu^2+^ does introduce two additional lower-energy excitations at around 3.8 and 4.7 eV on the regular surface, although the intensity of these peaks is relatively low. One could then reasonably expect that these perturbations will merge into the more sizeable collective excitations of the metal clusters, as indeed observed and discussed in the next subsection. Thus, we can expect these doping effects to become important only when a QM mixing of cluster and support electronic states takes place in semi-conducting oxides. Finally, when we put a Cu^2+^-cation replacement next to an oxygen vacancy, we observe a significant interaction of these two point defects, with a broadening, red-shifting, and a fragmentation of the excitations down to ≈ 2.0 eV: the two additional excitations at ≈ 3.8 and 4.7 eV are fragmented and added by a series of smaller peaks at ≈ 2.0 eV and between ≈ 2.6 and 3.4 eV when the MgO surface exhibits an oxygen vacancy simultaneously with a Cu^2+^-cation replacement, see Figure 2b. As we will see in the next subsection, these phenomena will interact in a subtle way with the cluster excited states.

Overall, from the results of this subsection, we can conclude that the accuracy of our computational approach is validated and found to be reasonably predictive, so that we can use it to explore unknown systems in the next subsection.

### 2.2. Optical Response of Cluster/Substrate Systems

As described in the section on “Materials and Methods”, we performed geometry relaxations (using the ORCA code) followed by TDDFT simulations (using different codes: ORCA, ADF, and NWChem), and we will focus on the results for the following systems:

- Ag20/MgO(100)-reg,
- Ag20/MgO(100)-Ovac,
- Ag20/MgO(100)-Cu,
- Ag20/MgO(100)-Ovac+Cu,
- Au20/MgO(100)-reg,
- Au20/MgO(100)-Ovac,
- Ag20/MgO(100)-Cu,
- Ag20/MgO(100)-Ovac+Cu.

**Figure 3:**
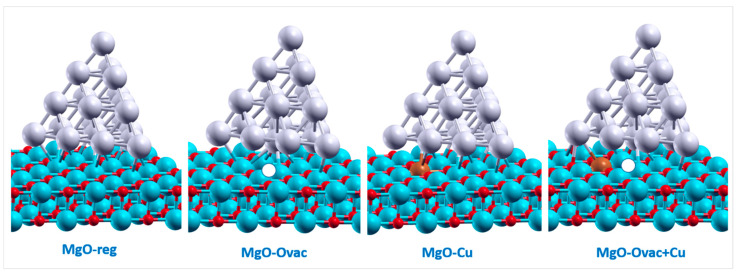
Representative geometries of some of the investigated systems—from left to right: Ag_20_/MgO(100)-reg, Ag_20_/MgO(100)-Ovac, Ag_20_/MgO(100)-Cu, and Ag_20_/MgO(100)-Ovac+Cu. Color coding: oxygen in red, magnesium in light blue, copper in brown, and oxygen vacancy represented with a white sphere.

The geometries of these systems are schematically depicted in Figure 3, as well as reported as Cartesian coordinates in the SM. Let us start the discussion of our results from the regular MgO(100) surface.

**Figure 4:**
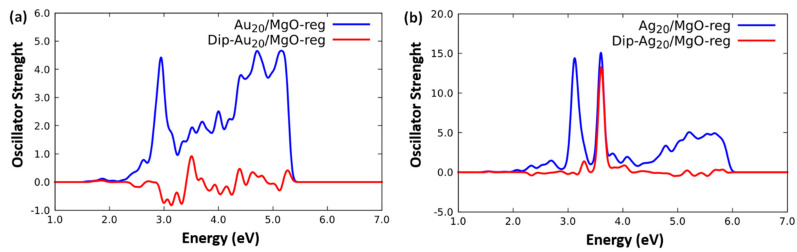
Blue curves: simulated TDDFT/PBE0 photo-absorption spectra of: (**a**) Au_20_/MgO(100)-reg and (**b**) Ag_20_/MgO(100)-reg using ORCA code. Red curves: signed contribution to the oscillator strengths associated with the *perpendicular* component of the transition dipole moment.

Figure 4 (blue curves) reports the corresponding Ag_20_ and Au_20_ spectra using the ORCA code. For comparison and to show the kind of cross-validation that it is possible to achieve in these systems, in Figure S4 of the supplementary materials, we also report the ADF results for the same systems. The slightly lower values of excitation energies found with the ADF code for Ag_20_ are due to minor computational differences in the two codes. By comparing Figure 1 and Figure 4, we find that the interaction with a charge-separated oxide support and the associated electric field leads to two effects.

First, a broadening and a fragmentation of the spectra. In both the Ag_20_ and Au_20_ cases, the symmetry-breaking produced by the oxide substrate introduces coupling matrix elements into the Casida response matrix, which mix the metal-cluster excitations, so as to make bright some of the excitations that were dark in the gas-phase due to symmetry selection rules. This effect is more apparent in the region between 3 and 4 eV of Au_20_, and in the fact that the major peak of Ag_20_ around 3.7 eV notably splits into two peaks down to ≈ 3.1 and ≈ 3.6 eV.

Second, a shift toward lower energies of the absorption peaks. Indeed, the two lowest-energy peaks of Au_20_ at ≈ 3.1 and ≈ 3.3 eV merge into a single broader one at around ≈ 2.8 eV, while as mentioned, the main Ag_20_ peak splits into two peaks, one of which is substantially red-shifted by 0.6 eV. This is due to the fact that some of the metal-cluster excitations have a transition dipole moment that interact favorably with the electric field due to the substrate and are thus stabilized (see below). Both these effects can be important in terms of applications of these systems as photo-enhancers or photochemical promoters because they extend the energy range of absorbed sunlight and bring it to cover a lower-energy part of the solar spectrum. These effects are qualitatively similar to what was previously observed for very small clusters (hexamers) [20], but are here quantitatively more important, and, interestingly, go in the direction of weakening the molecular-like character of the response to become more similar to pre-plasmonic excitations. The difference between Ag_20_ and Au_20_ spectra can be explained in terms of a stronger chemical interaction between Au atoms and oxygen anions with respect to Ag atoms (see the data on adsorption energies reported in the SM), due to a larger admixture of *d*-orbitals into the metal(M)-surface bonding [29], although this does not transpire in the M-O interface distances that range in both cases in the interval 2.5–2.8 Å.

It is important to underline that these effects are not due to structural (geometric) reasons but only to electrostatic and wave-function-deformation effects: this is proven by the fact that, e.g., the spectra of Ag_20_ in the geometry extracted from the Ag_20_/MgO(100)-reg interacting system, reported in Figure S3 of the SM, does not show any significant difference with respect to the spectrum of gas-phase Ag_20_.

We now focus on analysis tools that can help to characterize the excitations and possibly identify their link to those appearing in larger systems. Looking forward to studying the interaction between the excitations of the aggregate and those of more complicated (reducible-oxide) supports, it is convenient to analyze the transition dipole moments of the excited states, and to distinguish the Cartesian components parallel and perpendicular to the surface. This will play a role in translating the present results to non-inert supports exhibiting a coupling between supported-particle and substrate excitations [20]. The perpendicular component should in fact favor the injection of charge into the substrate, whereas the parallel component may give rise to energy transfer effects with correspondingly oriented substrate excitations. Figure 4 (red curves) then reports the signed contribution to the oscillator strengths associated with the perpendicular component of the transition dipole moment. Note that the parallel component can be deduced as the algebraic difference of the total oscillator strength and the modulus of the signed perpendicular contribution. Interestingly, for Ag_20_ the lower-energy peak at ≈ 3.1 is essentially parallel to the substrate, whereas the higher-energy peak ≈ 3.6 eV is essentially perpendicular to the substrate. For Au_20_, the situation is more complex, but equally interesting: in this case the parallel component is mostly dominant, except in the region between ≈ 2.9 and ≈ 3.6 eV in which it amounts up to ≈ ½ (half) of the intensity, changing its sign going across it. The atomistic structure of these tetrahedral clusters clearly plays a role here, together with a more general phenomenon associated with the stronger chemical interaction with the surface of Au with respect to Ag (see the data on adsorption energies reported in the SM) that can be expected also in larger systems.

**Figure 5:**
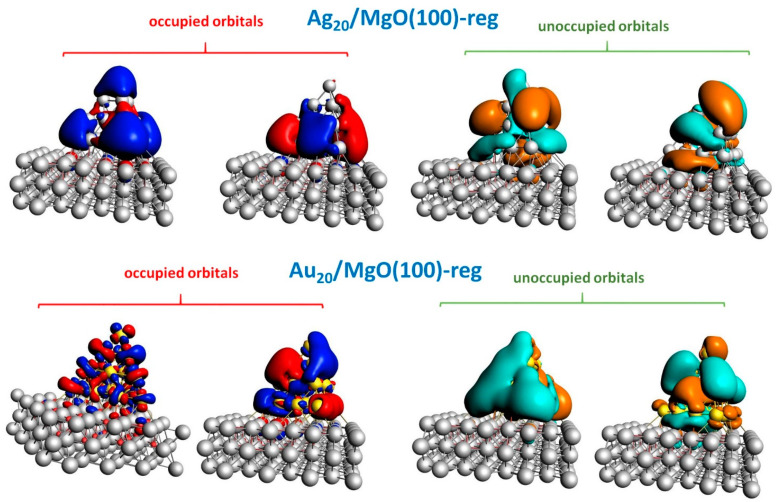
Contour plots of the Molecular Orbitals (MOs) of Ag_20_/MgO(100)-reg and Au_20_/MgO(100)-reg systems simulated using the ADF code. MO energy increases from left to right. Isosurfaces are set to contour values of 0.01 Å^−3/2^.

To gain further insight, we plot in Figure 5 the Molecular Orbitals (MOs) of Ag_20_/MgO(100)-reg and Au_20_/MgO(100)-reg most involved in the main lowest-energy absorption peaks (note that MO energies increase from left to right). A visual inspection of Figure 5 provides clarifying information. First, one can immediately appreciate the more atomic-like appearance of the occupied MOs of Au_20_/MgO(100)-reg with respect to those of Ag_20_/MgO(100)-reg. This is due to the more pronounced molecular-like character of the MOs in Au_20_ with respect to Ag_20_ due to the coupling between *s*- and *d*-band excitations in gold-based systems [24,25]. This is also the reason for the smaller intensity of its photo-absorption peaks, as recalled above. Second, one can observe that in both Ag_20_/MgO(100)-reg and Au_20_/MgO(100)-reg the higher-energy unoccupied (virtual) MO points outwards from the MgO100) surface, whereas the lower-energy unoccupied (virtual) MO points parallel to the surface. This explains why the perpendicular component of the transition dipole moment (red curves in Figure 4) prevails in the higher-energy peaks and not in the lower-energy peaks. One can additionally observe that this effect is more pronounced for Ag_20_, in agreement with the clearer distinction of the perpendicular contribution to the two split peaks in Figure 4.

**Figure 6:**
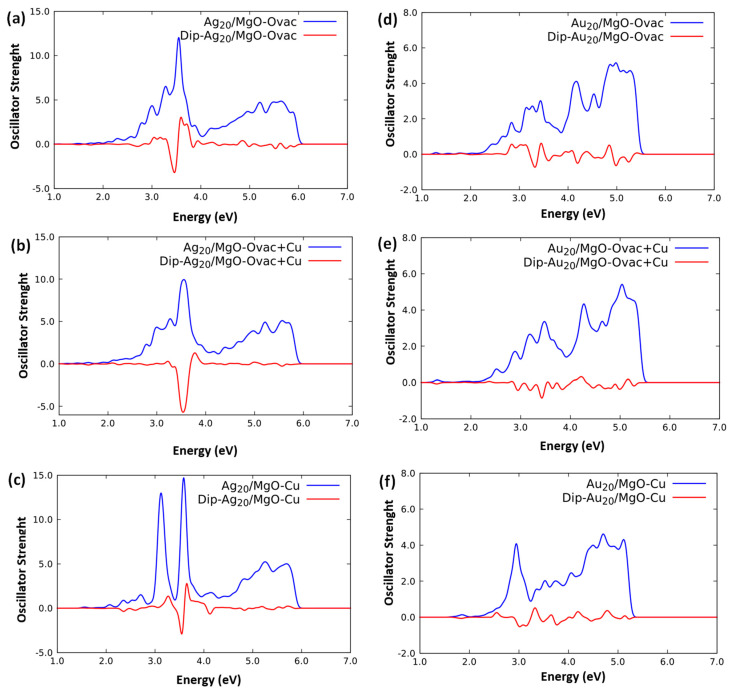
Blue curves: simulated TDDFT/PBE0 photo-absorption spectra of: (**a**) Ag_20_/MgO(100)-Ovac, (**b**) Ag_20_/MgO(100)-Ovac+Cu, (**c**) Ag_20_/MgO(100)-Cu, (**d**) Au_20_/MgO(100)-Ovac, (**e**) Ag_20_/MgO(100)-Ovac+Cu, (**f**) Ag_20_/MgO(100)-Cu, using the ORCA code. Red curves: signed contribution to the oscillator strengths associated with the perpendicular component of the transition dipole moment.

Figure 6 reports the spectra of Ag_20_/MgO(100)-Ovac–Figure 6a, and Au_20_/MgO(100)-Ovac–Figure 6d, obtained using the ORCA code. The most important conclusion that can be drawn from an inspection of these spectra is that in both cases the spectral broadening already observed for the regular surface is also present here, and even intensified. The final effect is, however, different for Ag_20_ and Au_20_. We can describe and explain this difference in terms of a “fragment” analysis of the free M_20_ cluster fragments interacting with the MgO(100)-Ovac surface fragment and thus producing the M_20_/MgO(100)-Ovac composite system, as follows. The lower-energy peak of supported Ag_20_ at ≈ 3.1 eV, generated by the interaction with the oxide surface as a split from the main plasmonic excitation of gas-phase Ag_20_, see Figure 4b, mixes in with the lowest-energy oxygen-vacancy excitation peak at ≈ 2.8 eV, see Figure 2a. In this mixing, Ag_20_ lends significant absorption intensity to the vacancy excitation but is “repelled” at higher energy (i.e., blue-shifted). In this process, the lower-energy peak loses some intensity, so that the strongest absorption in the spectrum becomes the higher-energy peak of supported Ag_20_, here slightly stabilized by the interaction with the oxygen vacancy to 3.5–6 eV. In the case of Au_20_, instead, the lowest-energy peak of the supported metal cluster at ≈ 2.9 eV, see Figure 4a, nearly overlaps with the intrinsic excitations of the vacancy around ≈ 2.8 eV, see Figure 2a. This leads to a substantial broadening of the peaks that in turn brings as a final result a further significant amount of oscillator strength into the higher part of the optical region, around 2.8 eV.

Focusing now on the analysis of the transition dipole moments of the excited states, whose signed perpendicular component is reported as a red curve in Figure 6a,d, we observe a very different trend with respect to the corresponding plots for the regular substrate. In Ag_20_ there is a switch in the parallel vs. perpendicular components of the main peaks reminiscent of the two split peaks of the Ag_20_/MgO(100), but here the higher-energy peak at 3.5–6 eV loses most of its perpendicular component in favor of the transition region around ≈ 3.3 eV (between ≈ 3.1 and ≈ 3.5 eV). At variance, for Au_20_ an overall weakening of the perpendicular component is observed.

**Figure 7:**
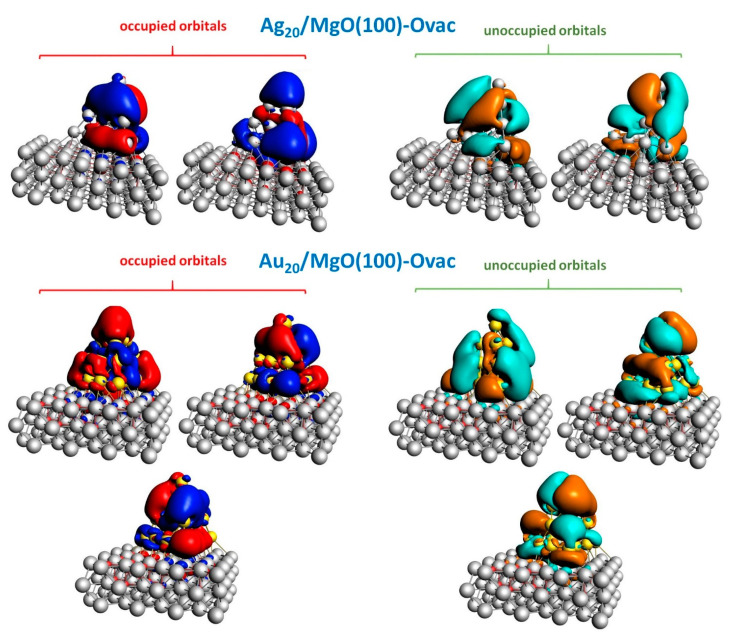
Contour plots of the Molecular Orbitals (MOs) of Ag_20_/MgO(100)-Ovac (**top-most row**) and Au_20_/MgO(100)-Ovac (**bottom two rows**) systems simulated using the ADF code. MO energy increases from left to right and (for the Au_20_ case) from top to bottom. Isosurfaces are set to contour values of 0.01 Å^−3/2^.

Plots of the Molecular Orbitals (MOs) of the Ag_20_/MgO(100)-Ovac and Au_20_/MgO(100)-Ovac systems most involved in the main lowest-energy absorption peaks in Figure 7 provide further insight.

In particular, for Ag_20_/MgO(100)-Ovac, by comparison with the corresponding MOs of the Ag_20_/MgO(100)-reg system (we repeat the MO plots in Figure S6 of the SM for convenience of the reader and an easier comparison) one can appreciate that the mixing between the MOs of the cluster and of the electrons localized in the vacancy inverts the character of the lower- and higher-energy occupied MOs, with the lower-energy occupied MO losing its symmetric shape. This explains the decrease in the perpendicular component to the higher-energy absorption peak. Furthermore, one can appreciate that the higher-energy occupied MO acquires a more global (slightly less fragmented) character, still mostly parallel to the surface but with a much stronger perpendicular component. This explains the increase in the perpendicular component in the transition region around ≈ 3.3 eV.

For Ag_20_/MgO(100)-Ovac, instead, the most notable phenomenon is the fragmentation of the lowest-energy unoccupied orbital, which does not couple any more via a dipole with the highest-energy occupied orbital, thus explaining the strong decrease in intensity in the lowest-energy spectral peak at ≈ 2.9 eV. The observations drawn from the inspection of the spectra are therefore nicely rationalized by this MO analysis.

Finally, in Figure 6, we also report the absorption spectrum of the Ag_20_/MgO(100)-Ovac+Cu, Figure 6b, Ag_20_/MgO(100)-Cu, Figure 6c, Au_20_/MgO(100)-Ovac+Cu, Figure 6e, and Au_20_/MgO(100)-Cu, Figure 6f, systems. Incidentally, for comparison and to check whether our conclusions might change when using transition metal dopants with a less filled *d*-shell, we have also simulated the TDDFT spectrum of the Ag_20_/MgO(100)-Ovac+Co system, i.e., a system in which we have doped one Mg^2+^ site with cobalt instead of copper—this spectrum is reported in Figure S5 of the SM, and is rather similar to that of the Ag_20_/MgO(100)-Ovac+Cu system. As anticipated in the previous subsection, the replacement of a Mg^2+^ cation with a transition metal analogue introduces very localized and quantitatively appreciable but not massive changes in the spectral profiles, whose effect on the metal cluster excitations in Ag_20_/MgO(100)-Cu and Au_20_/MgO(100)-Cu is hardly visible because it merges into and is obscured by their quantitatively larger peaks and the effect induced by the ionic substrate. These effects may be expected to become important only when analyzing the precise QM mixing of the electronic states of the cluster and a reducible oxide support. For the same reasons, we do not find a significant difference between the Cu^2+^ and Co^2+^ substitution (see Figure S5 of the SM). Analogously, when combining an oxygen vacancy and a Cu^2+^-replacement point defect in Ag_20_/MgO(100)-Ovac+Cu and Au_20_/MgO(100)-Ovac+Cu, we do not find large changes in the absorption profile with respect to Ag_20_/MgO(100)-Ovac and Au_20_/MgO(100)-Ovac: only an overall further broadening and a rounding of the main peaks can be observed.

However, the analysis of the transition dipole moments of the excited states in Figure 6 (red curves) provides additional and unexpected information. For the Ag case, we observe an opposite behavior for the separate Cu^2+^-replacement or oxygen vacancy defects with respect to when they are next to each other. In Ag_20_/MgO(100)-Cu, in fact, we can observe in Figure 6c a strong decrease in the perpendicular component of the higher-energy peak. In contrast, in Ag_20_/MgO(100)-Ovac+Cu, the higher-energy peak at ≈ 3.6 eV becomes substantially dominated by the perpendicular component, at a much higher degree than in either Ag_20_/MgO(100)-Ovac or Ag_20_/MgO(100)-Cu. We can thus conclude that, although the absorption profile is not substantially affected by combining an oxygen vacancy and a Cu^2+^-replacement point defect in Ag_20_/MgO(100)-Ovac+Cu, the proximity of these two point defects does change the shape of the excitations and can strongly favor the injection of charge into the substrate for the higher-energy excitations, while in general, energy-transfer processes between cluster and substrate excitations oriented in parallel are expected to be dominant. In the Au case, instead, the trend is different. Both the spectra of Au_20_/MgO(100)-reg and Au_20_/MgO(100)-Ovac are dominated by the parallel component. The introduction of a Cu^2+^-replacement diminishes this component, but only slightly. In contrast, combining an oxygen vacancy and a Cu^2+^-replacement point defect in Au_20_/MgO(100)-Ovac+Cu basically completely annihilates the perpendicular component in favor of the parallel one. It thus seems that the Ag systems with their more pronounced plasmonic character may give rise to more interesting QM-mixing phenomena than the *s*/*d*-coupling-damped Au systems.

## 3. Materials and Methods

As discussed in the introduction, to correctly capture particle/substrate interface effects in the optical excitations of particle/substrate composite systems, we need to reach an atomistic and quantum-mechanical (QM) level of theory. We thus aim at conducting atomistic simulations using time-dependent density-functional theory (TDDFT) [30], which represents the best compromise between accuracy and computational effort when dealing with excited states of molecular and solid-state systems [31,32]. In TDDFT, which is a linear response method grounded on the self-consistent solution of the density-functional theory (DFT) Kohn–Sham equations, one needs to select two exchange-correlation (xc-)functionals: the one employed in the Kohn–Sham equations and the one employed in the TDDFT response kernel. Here we selected in both cases a hybrid xc-functional, i.e., containing a mixture of the Hartree–Fock non-local exchange, the PBE0 xc-functional [33]. Since we do not have available software that implements periodic boundary conditions and is able to deal with hybrid xc-functionals in the response, we are led to use a finite-cluster model. In previous studies [20,34,35], we optimized the construction of a finite cluster of MgO(100) that can be used to reproduce as closely as possible the properties of species adsorbed on it (including metal clusters). In this finite-cluster modeling, only a small part of the MgO system is explicitly described at the QM level, i.e., those atoms that take part directly in the optical excitation process or that are anyway close to the excitation centers, while a large ensemble of point charges (q = +2 e for the Mg^2+^ cations and q = −2 e for the O^2−^ anions) allows us to accurately represent the Madelung potential generated by the MgO(100) surface, yet representing a minor part of the computational effort, since point charges only enter one-electron integrals. As previously discussed [34], a delicate point is how to deal with the boundary between the QM part and the point charges. In particular, all the point charges mimicking Mg^2+^ cations at the interface with the explicitly QM atoms are added of either pseudopotential or explicit (frozen) wave functions to avoid unphysical energy terms due to their interaction with the tails of the orbitals on the QM-described O^2−^ anions. Previously established rules [34] foresee that, for an accurate reproduction of the energetics, (i) all the atoms in the support at a bonding distance from the adsorbates, and additionally all their first-neighbors, be included into the QM region, that (ii) all the explicitly-QM atoms must be at least 3 MgO units far from the boundary of the finite cluster model, and that (iii) one should avoid polar borders or any uncompensated electrostatic contribution, which may give rise to electrostatic divergences [36]. The final finite-cluster model including explicitly: QM atoms, pseudopotential or simplified Mg^2+^ cations, and point charges is pictorially illustrated in Figure S1 of the Supplementary Materials (SM): the number of atoms in each group are 72, 60 and 3324, respectively. This approach is sufficient to make the DFT wave function of the adsorbates coincide with that derived from periodic boundary conditions (in which there is no QM/MM interface) [34,37]. However, here we found that, when studying excited states of this composite and large system, further special care is needed, because the QM-described O^2−^ anions at the interface with the MM region can have a spurious electronic polarization or deformation, which can spoil the optical spectrum with unphysical intruder states (these larger systems are even more prone to such issues than the minimal system investigated in Reference [20]). For example, we found that approximated schemes, such as the Hybrid Diagonal Approximation [27], produce results of lower quality than in standard fully-QM molecular systems so far investigated precisely because of these boundary issues. To achieve robust predictions, we have used and contrasted the results of three different codes: ORCA [38,39], ADF [40,41] and NWChem [42], where ORCA has been used for production, while ADF and NWChem have been used for cross-validation. We have shown in the previous section an example of comparison and cross-validation between ORCA and ADF. The computational details of the three codes are as follows. ORCA ground state and excited state energetics were evaluated employing def2-TZVP basis sets on transition metal atoms and def2-SVP basis sets on Mg and O centers [43]. To reduce the computational burden, Coulomb and Exchange integrals were evaluated by using respectively the Resolution of the Identity (RI) [44] and the Chain of Spheres (COSX) approximations [45] along with def2/JK auxiliary basis sets. For ADF, an all-electron basis set consisting of Slater Type Orbitals (STO) of TZP quality was employed, and relativistic effects were treated within the Zero Order Regular Approximation (ZORA) formalism at the Scalar Relativistic (SR) level. For NWChem, we used the same computational set-up as in Reference [20]. In ORCA and NWChem, the hybrid Mg^2+^ cations at the interface were described with centers in which a repulsive pseudopotential is positioned in addition to a q = +2 charge (see Reference [34] and references therein), whereas in ADF a frozen-core Mg^2+^ wave function was used in addition to a q = +2 charge. Finally, to simulate the MgO(100) surface exhibiting a neutral oxygen vacancy [21], we removed an oxygen nucleus from the system and kept the total charge of the system neutral, corresponding to having two electrons localized in the vacancy site left by removal of the oxygen nucleus and stabilized by the Madelung potential of the ionic MgO(100) surface.

The optical spectra were simulated by performing TDDFT simulations on relaxed geometries, which were obtained using the CP2K code [46] and the Perdew–Burke–Ernzerhof (PBE) xc-functional [47], augmented with van-der-Waals Grimme-D3 dispersion terms [48]. The oxide support of our models was a periodic three-layer thick 4 × 4 MgO(100) supercell, whose atoms were kept frozen at the experimental distances during relaxations. Valence electrons were described using DVZP-MOLOPT basis sets [49], whereas the core electrons were substituted with GTH pseudopotentials [50]. An auxiliary plane wave basis set with a cutoff of 300 Ry was employed to obtain the periodic Hartree term of the DFT equations. Note the use of a different xc-functional in geometry relaxation than in optical excitation simulations because of the different physics in these processes. We have previously validated in fact that PBE + dispersion optimally reproduces energy and geometry results on similar systems from a very sophisticated exact-exchange-plus-correlation approach in the random phase approximation (EX-cRPA/cRPA+) simulations [51]. Once the geometry is properly predicted, it has also been shown in previous work that a hybrid xc-functional is necessary to obtain accurate predictions of the optical spectra of metal clusters [24,25,52], as well as molecular systems [31].

## 4. Conclusions

In the present work, we aimed at exploring the optical (plasmonic) response of oxide-supported metal particles via rigorous time-dependent density-functional theory (TDDFT) simulations. As models of metal particles, we selected two clusters—Ag_20_ and Au_20_—that are small enough to be amenable to Quantum-Mechanical (QM) TDDFT simulations and have widely been employed to represent plasmonic metal particles [22,23,24]. Ag_20_ and Au_20_ indeed exhibit strong absorption peaks (although in the near-UV rather than optical region), which can correlate with the Localized Surface Plasmon Resonances (LSPR) of larger systems. As models of the oxide substrate, we chose the MgO(100) surface, both regular and exhibiting examples of point defects: an oxygen vacancy on the anion framework or substitutional transition-metal dopants (Cu^++^ or Co^++^ replacing Mg^++^) on the cation framework. The MgO support, being a simple wide-band-gap oxide, allowed us to use finite-cluster models for the support while keeping accuracy under control, and to disentangle the effects due to electrostatic perturbation of the metal excitations from those associated with QM mixing of cluster and support electronic states.

Our main conclusions are as follows.

First, technically, the present protocol seems able to treat accurately realistic systems with an affordable computational effort.

Second, in terms of results, we predict that the interaction with a charge-separated oxide substrate will significantly alter the optical response of these systems, as drawn from an inspection of the simulated spectra and their perpendicular vs. parallel components of the transition moment dipole and rationalized via an analysis of the system’s Molecular Orbitals (MOs). Even in the case in which there is the least mixing of the cluster/support wave functions, i.e., the MgO(100) regular surface, we predict an appreciable perturbation of the spectra. For example, the pre-plasmonic peak of Ag_20_ splits into two peaks: a lower-energy one exhibiting a transition dipole moment parallel to the surface and red-shifted by 0.6 eV, and a higher-energy peak only slightly energy-stabilized and exhibiting a transition dipole moment perpendicular to the surface. The main peaks of Au_20_ in the near-UV also see a stabilization by 0.4–0.5 eV into the optical region. This red-shifting effect is accompanied by a broadening and a fragmentation of the absorption peaks. These broadening and fragmentation phenomena are particularly apparent when a point defect is present in the oxide such as an oxygen vacancy with its highly polarizable electronic states coupled and mixed with the metal cluster excitations. We also considered local doping of the cation framework with transition metal impurities, and, unexpectedly, we found that, in the Ag_20_ case, the co-presence of this defect and of a neighboring oxygen vacancy can induce mixing of the electronic excited-state wave functions that change the character (parallel or perpendicular to the surface) of the optical response of the metal clusters, thus being of interest in view of charge injection or energy-transfer phenomena. An opposite behavior was instead found in the Au_20_ case.

In terms of perspectives, the extension of this work to reducible oxides seems the most promising direction. Further work may also focus on larger systems closer to the photocatalytic nanoparticles typically used in experiments. The use of analysis tools such as the Individual Component Map of Oscillatory Strength (ICM-OS) plots [53] as well as the extension to chiral systems, including the use of analysis tools such as the Individual Component Map of Oscillatory Strength (ICM-RS) plots [54] could turn out to be very useful when investigating larger systems.

## Data Availability

The data presented in this study are available in the Supplementary Material. Further data such as output files are available on request from the corresponding authors. These latter data are not publicly available due to the huge size of the files and since they are not necessary to reproduce the results.

## Supplementary Materials

The following are available online. The Supplementary Materials (SM) include: Figure S1. Pictorial illustration of the decomposition of the finite-cluster model used to model the substrate, including explicitly QM atoms (QM), pseudopotential or simplified Mg^2+^ cations (Boundary), and point charges (Point Charges); Figure S2. Representative geometries of the fragment systems (see Section 2.1 of the main text): (a) Ag_20_, and (b) the QM model of MgO(100) with a Mg^2+^ → Cu^2+^ replacement next to an oxygen vacancy (color coding: oxygen in red, magnesium in light blue, copper in brown); Figure S3. Simulated TDDFT/PBE0 spectrum of Ag_20_ in various geometries: gas-phase (blue curve), extracted from the Ag_20_/MgO(100)-reg interacting system (red curve), and extracted from the Ag_20_/MgO(100)-Ovac interacting system (green curve); Figure S4. Comparison and cross-validation between simulated TDDFT/PBE0 spectra of: (a) Ag_20_/MgO(100)-reg and (b) Au20/MgO(100)-reg using the ORCA (blue curves) and ADF (red curves) codes; Figure S5. Comparison of the simulated TDDFT/PBE0 spectra of the Ag_20_/MgO(100)-Cu and Ag_20_/MgO(100)-Co systems using the ORCA code; Figure S6. Contour plots of the Molecular Orbitals (MOs) of the Ag_20_/MgO(100)-reg and Ag_20_/MgO(100)-Ovac systems using the ADF code. Isosurfaces are set to contour values of 0.01 Å^−3/2^. These plots are taken for convenience of the reader and an easier comparison from Figure 5 and Figure 7 of the main text.
