# Supplementary material for: Optical Activity of Metal Nanoclusters Deposited on Regular and Doped Oxide Supports from First-Principles Simulations
Source: Molecules. 2021 Nov 18;26(22):6961. doi: 10.3390/molecules26226961 (PMC8624987; doi:10.3390/molecules26226961)
Supplement: Supplementary file 1 [file molecules-26-06961-s001.zip › molecules-1427304-Supplementary.pdf]

Supplementary Materials

Optical Activity of Metal Nanoclusters Deposited on Regular and Doped Oxide Supports from First-principles Simulations

Luca Sementa [1,*], Mauro Stener [2,*], Alessandro Fortunelli [3,*]

1 CNR- IPCF, Consiglio Nazionale delle Ricerche, 56124 Pisa, Italy
2 Dipartimento di Scienze Chimiche e Farmaceutiche, Università di Trieste, I-34127 Trieste, Italy
3 CNR-ICCOM, Consiglio Nazionale delle Ricerche, 56124 Pisa, Italy
* Correspondence: luca.sementa@cnr.it (L.S.); stener@units.it (M.S.); alessandro.fortunelli@cnr.it (A.F.)

Here we present further information complementing that one provided in the main text.

We first furnish some details on the energetics of the structural models.

To help the reader understand details of our approach, we also provide pictorial illustrations of the finite-cluster models for the support and of the fragment systems.

We then give a comparison and cross-validation of the simulated TDDFT/PBE0 spectra, using different codes.

We finally include the Cartesian coordinated of the relaxed geometries here considered, so that the simulations can be reproduced by others.

Starting with the energetics of the structural models, we first report the adsorption energies of the clusters. Defining the adsorption energy $E_{ads}(M_{20}/MgO)$ of a M_{20} cluster onto the MgO(100) slab as:

$$E_{ads}(M_{20}/MgO) = E(M_{20}/MgO) - E(M_{20}) - E(MgO) \qquad \text{(SM1)}$$

where $E(M_{20}/MgO)$ is the total energy of the composite cluster-on-MgO(100) system, $E(M_{20})$ is the energy of the relaxed M_{20} cluster in the gas phase, and E(MgO) is the energy of the MgO(100) slab, we find: $E_{ads}(Ag_{20}/MgO)$ = 4.80 eV, $E_{ads}(Au_{20}/MgO)$ = 5.95 eV. Using analogous definitions, for the MgO(100)-Ovac defected support we find: $E_{ads}(Ag_{20}/MgO\text{-}Ovac)$ = 5.82 eV, $E_{ads}(Au_{20}/MgO\text{-}Ovac)$ = 8.36 eV. In these values, the D3-estimated dispersion component is ≈3.13 eV for the Ag systems and ≈3.32 eV for the Au systems, respectively. These values of adsorption energies are sizeable, but are not sufficient to perturb the tough MgO(100) support significantly, as we showed in previous work by comparison of these finite-cluster simulations with the results of periodic systems in which the support was

allowed to relax [1,2]. It can also be recalled (see Figure S3) that the structural deformation induced by the interaction with the support does not change appreciably the spectrum of the free clusters. All this justifies the choice of our structural model.

1. Barcaro, G.; Fortunelli, A. The Interaction of Coinage Metal Clusters with the MgO(100) Surface. *J. Chem. Theory Comput.*, **2005**, *1*, 972–985, DOI: 10.1021/ct050073e.
2. Barcaro, G.; Edoardo Aprà, E.; Fortunelli, A. Structure of Ag Clusters Grown on Fs-Defect Sites of an MgO(100) Surface. *Chem. Eur. J.*, **2007**, *13*, 6408–6418, DOI: 10.1002/chem.200601796.

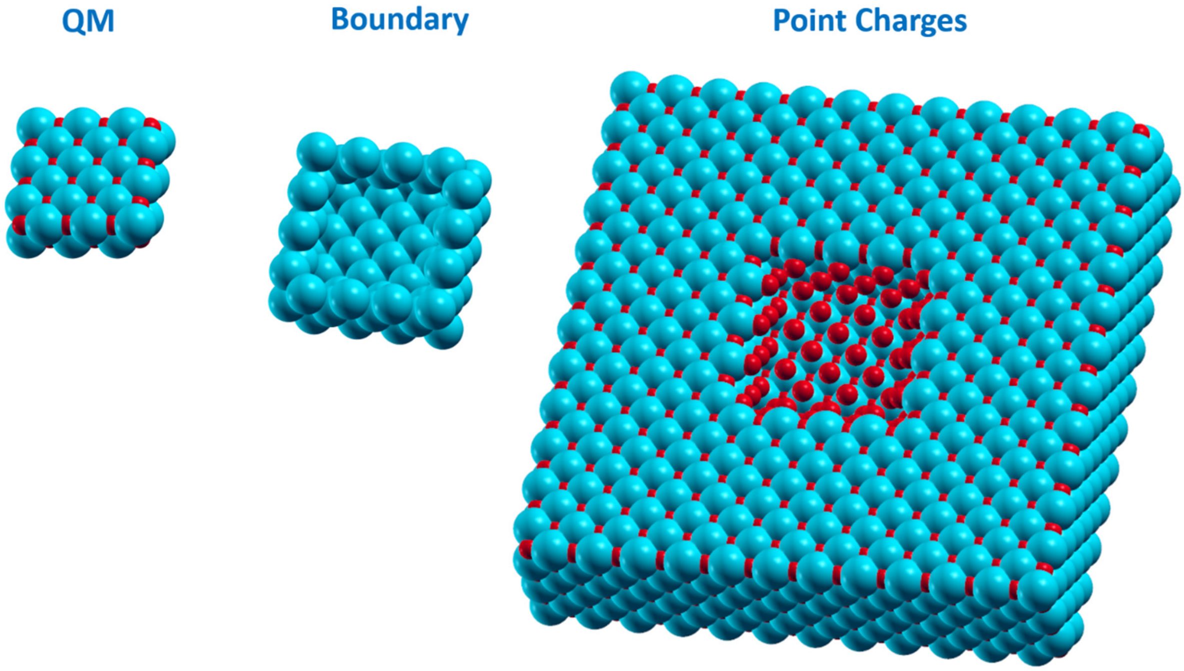

Figure S1. Pictorial illustration of the decomposition of the finite-cluster model used to model the substrate, including explicitly QM atoms (QM), pseudopotential or simplified Mg^{2+} cations (Boundary), and point charges (Point Charges).

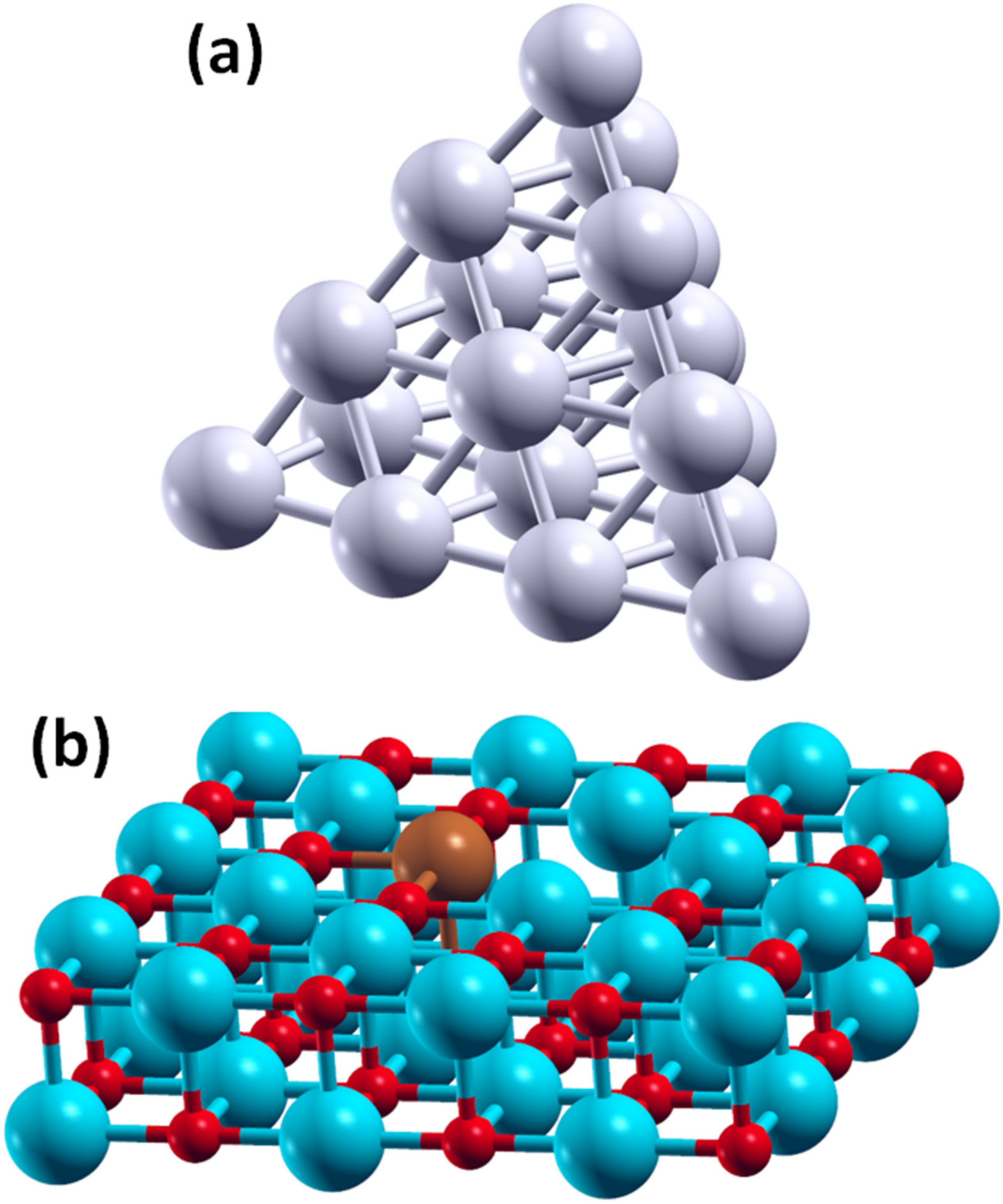

Figure S2. Representative geometries of the fragment systems (see Section 2.1 of the main text): (**a**) Ag_{20}, and (**b**) the QM model of MgO(100) with a $Mg^{2+} \rightarrow Cu^{2+}$ replacement next to an oxygen vacancy (color coding: oxygen in red, magnesium in light blue, copper in brown).

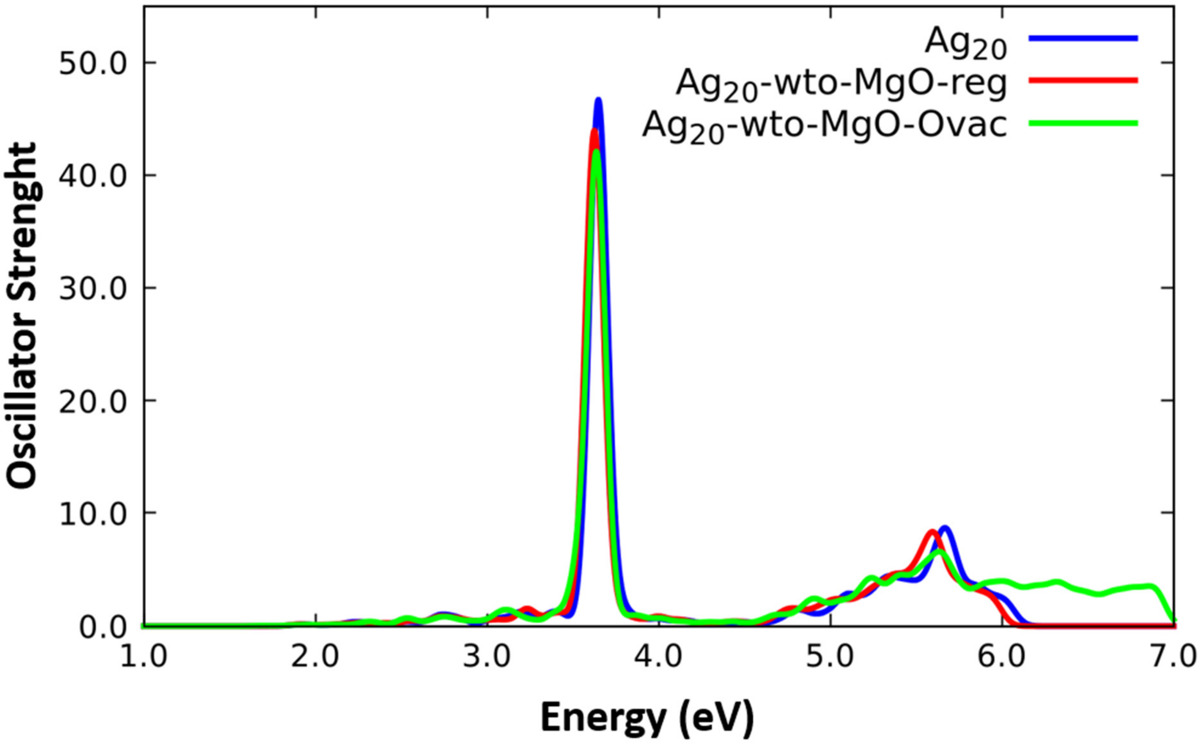

Figure S3. Simulated TDDFT/PBE0 spectrum of Ag_{20} in various geometries: gas-phase (blue curve), extracted from the Ag_{20}/MgO(100)-reg interacting system (red curve), and extracted from the Ag_{20}/MgO(100)-Ovac interacting system (green curve).

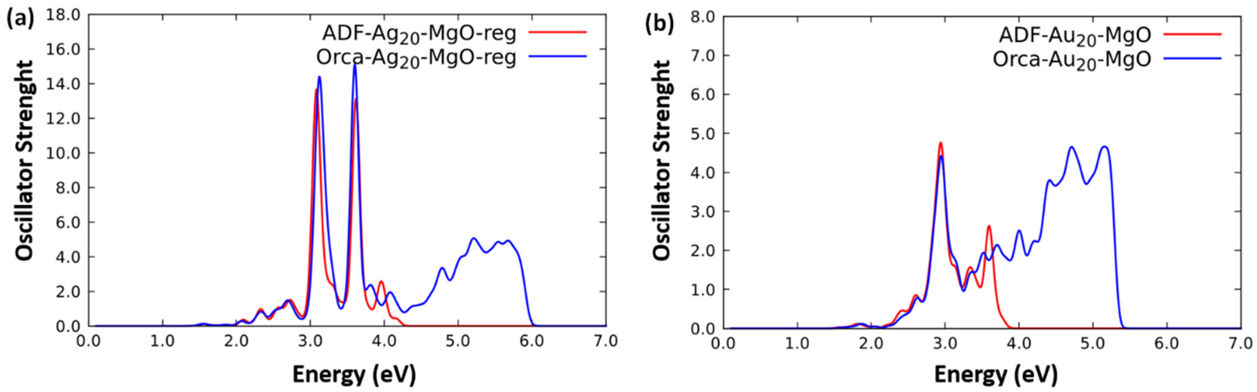

Figure S4. Comparison and cross-validation between simulated TDDFT/PBE0 spectra of: (**a**) Ag_{20}/MgO(100)-reg and (**b**) Au_{20}/MgO(100)-reg using the ORCA (blue curves) and ADF (red curves) codes.

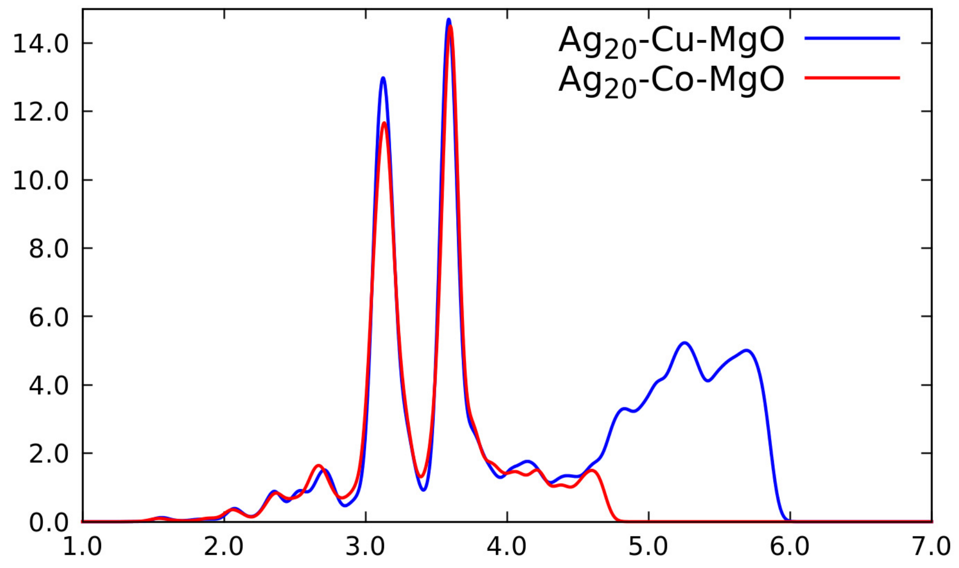

Figure S5. Comparison of the simulated TDDFT/PBE0 spectra of the Ag_{20}/MgO(100)-Cu and Ag_{20}/MgO(100)-Co systems using the ORCA code.

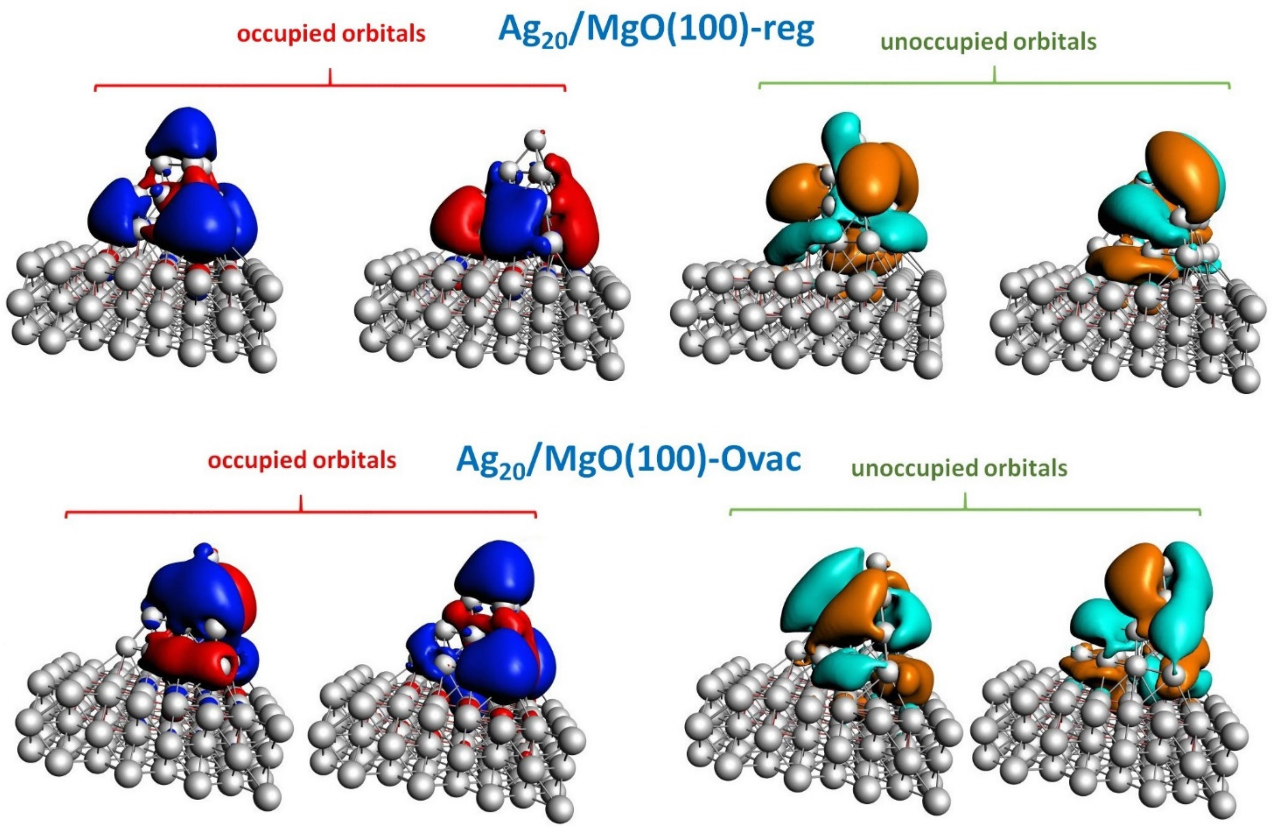

Figure S6. Contour plots of the Molecular Orbitals (MOs) of the Ag_{20}/MgO(100)-reg and Ag_{20}/MgO(100)-Ovac systems using the ADF code. Isosurfaces are set to contour values of 0.01 Å$^{-3/2}$. These plots are taken for convenience of the reader and an easier comparison from Figure 5 and 7 of the main text.

Cartesian Coordinates of Ag_{20}

Ag	12.3958991179	11.5473929146	4.7281748462
Ag	7.8754293801	6.4852929482	7.0386373102
Ag	9.0761505122	7.9370688692	11.4802588934
Ag	10.2035449568	9.1722070953	9.2677468237
Ag	11.4000134546	10.2732181654	7.0154911060
Ag	9.5559946196	6.2969862465	9.3080685597
Ag	10.8276995161	7.5007804143	7.0653798507
Ag	11.9496922913	8.6617108323	4.7141059015
Ag	10.1020620637	4.7322919408	7.0919386765
Ag	11.1335426743	5.9710570758	4.7270860783
Ag	10.6525512583	3.1676720237	4.8062628672
Ag	7.4218354405	8.2337402897	9.2462405368
Ag	8.5443230426	9.6084881584	7.0343937609
Ag	9.7303919250	10.6419907038	4.7085952487
Ag	9.0613712363	7.8808521025	4.7484771911
Ag	8.4542963026	4.8926458450	4.7365496581
Ag	5.9055381979	8.4995170891	6.9399142908
Ag	6.9908674476	9.8961151119	4.6747076311
Ag	6.4515287570	6.7602949863	4.6353965236
Ag	4.4339503807	8.6308159158	4.6252353688

Cartesian Coordinates of Ag_{20}-MgO-reg

Ag	12.3979913721	11.5565295413	4.7249375968
Ag	7.8925217666	6.4711935264	7.0400656289
Ag	9.0829547090	7.8760760151	11.4753871443
Ag	10.2292159453	9.1562267530	9.2774686230
Ag	11.4114027970	10.2767609896	7.0191039576
Ag	9.5987598881	6.2850064522	9.2844774997
Ag	10.8083203672	7.5101567929	7.0348503147
Ag	11.9394535057	8.6762416628	4.7041176411
Ag	10.1205177814	4.7297259239	7.0566127275
Ag	11.0876798135	6.0049705717	4.6944785384
Ag	10.6648123018	3.1678504198	4.7832795980
Ag	7.4376007881	8.2235105749	9.2461906594
Ag	8.5787360485	9.6079443512	7.0446229423
Ag	9.7395715648	10.6397826265	4.7027060790
Ag	8.9739297613	7.8932009067	4.7214104813
Ag	8.4417224411	4.8512939685	4.7283547982
Ag	5.9326285272	8.4960690954	6.9354879865
Ag	6.9820726192	9.9927524973	4.7267171066
Ag	6.3889775970	6.6600932913	4.6890163671
Ag	4.4566458809	8.6298289643	4.6259978609
Mg	8.42200000	6.31650000	2.10550000
Mg	8.42200000	2.10550000	2.10550000
Mg	8.42200000	10.52750000	2.10550000
Mg	6.31650000	8.42200000	2.10550000
Mg	6.31650000	4.21100000	2.10550000
Mg	6.31650000	12.63300000	2.10550000
Mg	4.21100000	6.31650000	2.10550000

Mg	4.21100000	2.10550000	2.10550000
Mg	4.21100000	10.52750000	2.10550000
Mg	2.10550000	8.42200000	2.10550000
Mg	2.10550000	4.21100000	2.10550000
Mg	2.10550000	12.63300000	2.10550000
Mg	12.63300000	6.31650000	2.10550000
Mg	12.63300000	2.10550000	2.10550000
Mg	12.63300000	10.52750000	2.10550000
Mg	10.52750000	8.42200000	2.10550000
Mg	10.52750000	4.21100000	2.10550000
Mg	10.52750000	12.63300000	2.10550000
Mg	8.42200000	8.42200000	0.00000000
Mg	8.42200000	4.21100000	0.00000000
Mg	8.42200000	12.63300000	0.00000000
Mg	6.31650000	6.31650000	0.00000000
Mg	6.31650000	2.10550000	0.00000000
Mg	6.31650000	10.52750000	0.00000000
Mg	4.21100000	8.42200000	0.00000000
Mg	4.21100000	4.21100000	0.00000000
Mg	4.21100000	12.63300000	0.00000000
Mg	2.10550000	6.31650000	0.00000000
Mg	2.10550000	2.10550000	0.00000000
Mg	2.10550000	10.52750000	0.00000000
Mg	12.63300000	8.42200000	0.00000000
Mg	12.63300000	4.21100000	0.00000000
Mg	12.63300000	12.63300000	0.00000000
Mg	10.52750000	6.31650000	0.00000000
Mg	10.52750000	2.10550000	0.00000000

Mg	10.52750000	10.52750000	0.00000000
O	8.42200000	8.42200000	2.10550000
O	8.42200000	4.21100000	2.10550000
O	8.42200000	12.63300000	2.10550000
O	6.31650000	6.31650000	2.10550000
O	6.31650000	2.10550000	2.10550000
O	6.31650000	10.52750000	2.10550000
O	4.21100000	8.42200000	2.10550000
O	4.21100000	4.21100000	2.10550000
O	4.21100000	12.63300000	2.10550000
O	2.10550000	6.31650000	2.10550000
O	2.10550000	2.10550000	2.10550000
O	2.10550000	10.52750000	2.10550000
O	12.63300000	8.42200000	2.10550000
O	12.63300000	4.21100000	2.10550000
O	12.63300000	12.63300000	2.10550000
O	10.52750000	6.31650000	2.10550000
O	10.52750000	2.10550000	2.10550000
O	10.52750000	10.52750000	2.10550000
O	8.42200000	6.31650000	0.00000000
O	8.42200000	2.10550000	0.00000000
O	8.42200000	10.52750000	0.00000000
O	6.31650000	8.42200000	0.00000000
O	6.31650000	4.21100000	0.00000000
O	6.31650000	12.63300000	0.00000000
O	4.21100000	6.31650000	0.00000000
O	4.21100000	2.10550000	0.00000000
O	4.21100000	10.52750000	0.00000000

O	2.10550000	8.42200000	0.00000000
O	2.10550000	4.21100000	0.00000000
O	2.10550000	12.63300000	0.00000000
O	12.63300000	6.31650000	0.00000000
O	12.63300000	2.10550000	0.00000000
O	12.63300000	10.52750000	0.00000000
O	10.52750000	8.42200000	0.00000000
O	10.52750000	4.21100000	0.00000000
O	10.52750000	12.63300000	0.00000000

Cartesian Coordinates of Ag_{20}-MgO-Ovac

Ag	12.3277568321	11.5978026346	4.7279126344
Ag	8.0067547972	6.5564905693	6.9386243739
Ag	9.0630633533	7.8836780723	11.4983833167
Ag	10.1688756398	9.1862627495	9.2964889240
Ag	11.3204369602	10.3665619186	7.0524359611
Ag	9.6097281053	6.3008837080	9.2892585252
Ag	10.7530383496	7.5838349100	7.0395571689
Ag	11.7484290794	8.7872250986	4.7186010913
Ag	10.1876339279	4.7931424715	7.0433266342
Ag	10.7206579710	6.2070357329	4.6417252428
Ag	10.7484817669	3.2506815151	4.7765117359
Ag	7.4332715806	8.1624729502	9.2387352873
Ag	8.5365167178	9.5277760430	6.9923775693
Ag	9.6232796310	10.8392717355	4.7974589370
Ag	8.6647642443	8.2267754357	4.1651213695
Ag	8.3473098998	4.6656101699	4.8018336749
Ag	5.9298362864	8.4490885715	6.9226454506
Ag	6.8448502370	10.2476635281	4.8186340478
Ag	6.3798844395	6.5666955092	4.7061889814
Ag	4.5012821003	8.6240952811	4.6129706617
Mg	8.42200000	6.31650000	2.10550000
Mg	8.42200000	2.10550000	2.10550000
Mg	8.42200000	10.52750000	2.10550000
Mg	6.31650000	8.42200000	2.10550000
Mg	6.31650000	4.21100000	2.10550000
Mg	6.31650000	12.63300000	2.10550000
Mg	4.21100000	6.31650000	2.10550000

Mg	4.21100000	2.10550000	2.10550000
Mg	4.21100000	10.52750000	2.10550000
Mg	2.10550000	8.42200000	2.10550000
Mg	2.10550000	4.21100000	2.10550000
Mg	2.10550000	12.63300000	2.10550000
Mg	12.63300000	6.31650000	2.10550000
Mg	12.63300000	2.10550000	2.10550000
Mg	12.63300000	10.52750000	2.10550000
Mg	10.52750000	8.42200000	2.10550000
Mg	10.52750000	4.21100000	2.10550000
Mg	10.52750000	12.63300000	2.10550000
Mg	8.42200000	8.42200000	0.00000000
Mg	8.42200000	4.21100000	0.00000000
Mg	8.42200000	12.63300000	0.00000000
Mg	6.31650000	6.31650000	0.00000000
Mg	6.31650000	2.10550000	0.00000000
Mg	6.31650000	10.52750000	0.00000000
Mg	4.21100000	8.42200000	0.00000000
Mg	4.21100000	4.21100000	0.00000000
Mg	4.21100000	12.63300000	0.00000000
Mg	2.10550000	6.31650000	0.00000000
Mg	2.10550000	2.10550000	0.00000000
Mg	2.10550000	10.52750000	0.00000000
Mg	12.63300000	8.42200000	0.00000000
Mg	12.63300000	4.21100000	0.00000000
Mg	12.63300000	12.63300000	0.00000000
Mg	10.52750000	6.31650000	0.00000000
Mg	10.52750000	2.10550000	0.00000000

Mg	10.52750000	10.52750000	0.00000000
O	8.42200000	4.21100000	2.10550000
O	8.42200000	12.63300000	2.10550000
O	6.31650000	6.31650000	2.10550000
O	6.31650000	2.10550000	2.10550000
O	6.31650000	10.52750000	2.10550000
O	4.21100000	8.42200000	2.10550000
O	4.21100000	4.21100000	2.10550000
O	4.21100000	12.63300000	2.10550000
O	2.10550000	6.31650000	2.10550000
O	2.10550000	2.10550000	2.10550000
O	2.10550000	10.52750000	2.10550000
O	12.63300000	8.42200000	2.10550000
O	12.63300000	4.21100000	2.10550000
O	12.63300000	12.63300000	2.10550000
O	10.52750000	6.31650000	2.10550000
O	10.52750000	2.10550000	2.10550000
O	10.52750000	10.52750000	2.10550000
O	8.42200000	6.31650000	0.00000000
O	8.42200000	2.10550000	0.00000000
O	8.42200000	10.52750000	0.00000000
O	6.31650000	8.42200000	0.00000000
O	6.31650000	4.21100000	0.00000000
O	6.31650000	12.63300000	0.00000000
O	4.21100000	6.31650000	0.00000000
O	4.21100000	2.10550000	0.00000000
O	4.21100000	10.52750000	0.00000000
O	2.10550000	8.42200000	0.00000000

O	2.10550000	4.21100000	0.00000000
O	2.10550000	12.63300000	0.00000000
O	12.63300000	6.31650000	0.00000000
O	12.63300000	2.10550000	0.00000000
O	12.63300000	10.52750000	0.00000000
O	10.52750000	8.42200000	0.00000000
O	10.52750000	4.21100000	0.00000000
O	10.52750000	12.63300000	0.00000000

Cartesian Coordinates of Ag_{20}-MgO-Cu

Ag	12.3958991179	11.5473929146	4.7281748462
Ag	7.8754293801	6.4852929482	7.0386373102
Ag	9.0761505122	7.9370688692	11.4802588934
Ag	10.2035449568	9.1722070953	9.2677468237
Ag	11.4000134546	10.2732181654	7.0154911060
Ag	9.5559946196	6.2969862465	9.3080685597
Ag	10.8276995161	7.5007804143	7.0653798507
Ag	11.9496922913	8.6617108323	4.7141059015
Ag	10.1020620637	4.7322919408	7.0919386765
Ag	11.1335426743	5.9710570758	4.7270860783
Ag	10.6525512583	3.1676720237	4.8062628672
Ag	7.4218354405	8.2337402897	9.2462405368
Ag	8.5443230426	9.6084881584	7.0343937609
Ag	9.7303919250	10.6419907038	4.7085952487
Ag	9.0613712363	7.8808521025	4.7484771911
Ag	8.4542963026	4.8926458450	4.7365496581
Ag	5.9055381979	8.4995170891	6.9399142908
Ag	6.9908674476	9.8961151119	4.6747076311
Ag	6.4515287570	6.7602949863	4.6353965236
Ag	4.4339503807	8.6308159158	4.6252353688
Mg	8.42200000	6.31650000	2.10550000
Mg	8.42200000	2.10550000	2.10550000
Mg	8.42200000	10.52750000	2.10550000
Cu	6.31650000	8.42200000	2.10550000
Mg	6.31650000	4.21100000	2.10550000
Mg	6.31650000	12.63300000	2.10550000
Mg	4.21100000	6.31650000	2.10550000

Mg	4.21100000	2.10550000	2.10550000
Mg	4.21100000	10.52750000	2.10550000
Mg	2.10550000	8.42200000	2.10550000
Mg	2.10550000	4.21100000	2.10550000
Mg	2.10550000	12.63300000	2.10550000
Mg	12.63300000	6.31650000	2.10550000
Mg	12.63300000	2.10550000	2.10550000
Mg	12.63300000	10.52750000	2.10550000
Mg	10.52750000	8.42200000	2.10550000
Mg	10.52750000	4.21100000	2.10550000
Mg	10.52750000	12.63300000	2.10550000
Mg	8.42200000	8.42200000	0.00000000
Mg	8.42200000	4.21100000	0.00000000
Mg	8.42200000	12.63300000	0.00000000
Mg	6.31650000	6.31650000	0.00000000
Mg	6.31650000	2.10550000	0.00000000
Mg	6.31650000	10.52750000	0.00000000
Mg	4.21100000	8.42200000	0.00000000
Mg	4.21100000	4.21100000	0.00000000
Mg	4.21100000	12.63300000	0.00000000
Mg	2.10550000	6.31650000	0.00000000
Mg	2.10550000	2.10550000	0.00000000
Mg	2.10550000	10.52750000	0.00000000
Mg	12.63300000	8.42200000	0.00000000
Mg	12.63300000	4.21100000	0.00000000
Mg	12.63300000	12.63300000	0.00000000
Mg	10.52750000	6.31650000	0.00000000
Mg	10.52750000	2.10550000	0.00000000

Mg	10.52750000	10.52750000	0.00000000
O	8.42200000	8.42200000	2.10550000
O	8.42200000	4.21100000	2.10550000
O	8.42200000	12.63300000	2.10550000
O	6.31650000	6.31650000	2.10550000
O	6.31650000	2.10550000	2.10550000
O	6.31650000	10.52750000	2.10550000
O	4.21100000	8.42200000	2.10550000
O	4.21100000	4.21100000	2.10550000
O	4.21100000	12.63300000	2.10550000
O	2.10550000	6.31650000	2.10550000
O	2.10550000	2.10550000	2.10550000
O	2.10550000	10.52750000	2.10550000
O	12.63300000	8.42200000	2.10550000
O	12.63300000	4.21100000	2.10550000
O	12.63300000	12.63300000	2.10550000
O	10.52750000	6.31650000	2.10550000
O	10.52750000	2.10550000	2.10550000
O	10.52750000	10.52750000	2.10550000
O	8.42200000	6.31650000	0.00000000
O	8.42200000	2.10550000	0.00000000
O	8.42200000	10.52750000	0.00000000
O	6.31650000	8.42200000	0.00000000
O	6.31650000	4.21100000	0.00000000
O	6.31650000	12.63300000	0.00000000
O	4.21100000	6.31650000	0.00000000
O	4.21100000	2.10550000	0.00000000
O	4.21100000	10.52750000	0.00000000

O	2.10550000	8.42200000	0.00000000
O	2.10550000	4.21100000	0.00000000
O	2.10550000	12.63300000	0.00000000
O	12.63300000	6.31650000	0.00000000
O	12.63300000	2.10550000	0.00000000
O	12.63300000	10.52750000	0.00000000
O	10.52750000	8.42200000	0.00000000
O	10.52750000	4.21100000	0.00000000
O	10.52750000	12.63300000	0.00000000

Cartesian Coordinates of Ag_{20}-MgO-Ovac-Cu

Ag	12.3281959739	11.5899243196	4.7261143136
Ag	7.9683555962	6.5369863672	6.8896622037
Ag	8.9974721170	7.8780194689	11.4570307501
Ag	10.1054842218	9.1966945785	9.2686376628
Ag	11.2970652441	10.3610507291	7.0375639030
Ag	9.5498564027	6.2915319848	9.2597817495
Ag	10.7472185824	7.5752259485	7.0456080298
Ag	11.7620322596	8.7754141528	4.7207585259
Ag	10.1724993303	4.7830954968	7.0309688804
Ag	10.7407683475	6.1842788325	4.6465212189
Ag	10.7517625022	3.2470576944	4.7622924465
Ag	7.3944684012	8.1584538335	9.1657571536
Ag	8.5021195049	9.5371120417	6.9510868141
Ag	9.6237275339	10.8233929695	4.7606222568
Ag	8.7029881840	8.1731759657	4.2470613034
Ag	8.3444221294	4.6508975392	4.7783818100
Ag	5.9023636887	8.4428673275	6.8343468764
Ag	6.8415856484	10.1901316425	4.7078600017
Ag	6.3883275330	6.5758391067	4.6161382019
Ag	4.5008427269	8.6100284422	4.5176769224
Mg	8.42200000	6.31650000	2.10550000
Mg	8.42200000	2.10550000	2.10550000
Mg	8.42200000	10.52750000	2.10550000
Cu	6.31650000	8.42200000	2.10550000
Mg	6.31650000	4.21100000	2.10550000
Mg	6.31650000	12.63300000	2.10550000
Mg	4.21100000	6.31650000	2.10550000

Mg	4.21100000	2.10550000	2.10550000
Mg	4.21100000	10.52750000	2.10550000
Mg	2.10550000	8.42200000	2.10550000
Mg	2.10550000	4.21100000	2.10550000
Mg	2.10550000	12.63300000	2.10550000
Mg	12.63300000	6.31650000	2.10550000
Mg	12.63300000	2.10550000	2.10550000
Mg	12.63300000	10.52750000	2.10550000
Mg	10.52750000	8.42200000	2.10550000
Mg	10.52750000	4.21100000	2.10550000
Mg	10.52750000	12.63300000	2.10550000
Mg	8.42200000	8.42200000	0.00000000
Mg	8.42200000	4.21100000	0.00000000
Mg	8.42200000	12.63300000	0.00000000
Mg	6.31650000	6.31650000	0.00000000
Mg	6.31650000	2.10550000	0.00000000
Mg	6.31650000	10.52750000	0.00000000
Mg	4.21100000	8.42200000	0.00000000
Mg	4.21100000	4.21100000	0.00000000
Mg	4.21100000	12.63300000	0.00000000
Mg	2.10550000	6.31650000	0.00000000
Mg	2.10550000	2.10550000	0.00000000
Mg	2.10550000	10.52750000	0.00000000
Mg	12.63300000	8.42200000	0.00000000
Mg	12.63300000	4.21100000	0.00000000
Mg	12.63300000	12.63300000	0.00000000
Mg	10.52750000	6.31650000	0.00000000
Mg	10.52750000	2.10550000	0.00000000

Mg	10.52750000	10.52750000	0.00000000
O	8.42200000	4.21100000	2.10550000
O	8.42200000	12.63300000	2.10550000
O	6.31650000	6.31650000	2.10550000
O	6.31650000	2.10550000	2.10550000
O	6.31650000	10.52750000	2.10550000
O	4.21100000	8.42200000	2.10550000
O	4.21100000	4.21100000	2.10550000
O	4.21100000	12.63300000	2.10550000
O	2.10550000	6.31650000	2.10550000
O	2.10550000	2.10550000	2.10550000
O	2.10550000	10.52750000	2.10550000
O	12.63300000	8.42200000	2.10550000
O	12.63300000	4.21100000	2.10550000
O	12.63300000	12.63300000	2.10550000
O	10.52750000	6.31650000	2.10550000
O	10.52750000	2.10550000	2.10550000
O	10.52750000	10.52750000	2.10550000
O	8.42200000	6.31650000	0.00000000
O	8.42200000	2.10550000	0.00000000
O	8.42200000	10.52750000	0.00000000
O	6.31650000	8.42200000	0.00000000
O	6.31650000	4.21100000	0.00000000
O	6.31650000	12.63300000	0.00000000
O	4.21100000	6.31650000	0.00000000
O	4.21100000	2.10550000	0.00000000
O	4.21100000	10.52750000	0.00000000
O	2.10550000	8.42200000	0.00000000

O	2.10550000	4.21100000	0.00000000
O	2.10550000	12.63300000	0.00000000
O	12.63300000	6.31650000	0.00000000
O	12.63300000	2.10550000	0.00000000
O	12.63300000	10.52750000	0.00000000
O	10.52750000	8.42200000	0.00000000
O	10.52750000	4.21100000	0.00000000
O	10.52750000	12.63300000	0.00000000

Cartesian Coordinates of Au_{20}

Au	12.3460935116	11.5190580511	4.6867969349
Au	7.8236578162	6.1850310359	7.0773789245
Au	9.2106764929	7.8216562807	11.3199023559
Au	10.3621009817	9.1543816284	9.1879013981
Au	11.5032587579	10.2478241040	6.9811559204
Au	9.7189611204	6.1478985527	9.2049728880
Au	11.1740183290	7.4087286676	7.1105254684
Au	12.0819823566	8.5882730840	4.6793285251
Au	10.2551304182	4.6081228480	7.0610991194
Au	11.4194991010	5.9138720198	4.7590757252
Au	10.7604737036	3.1582445842	4.7482489440
Au	7.4681846166	8.0852345617	9.1719652961
Au	8.5544334422	9.6304002089	7.0664180586
Au	9.7108853613	10.5368050300	4.6820449990
Au	9.1614991231	7.7035806976	4.8930857221
Au	8.4855955492	4.7619337658	4.7193463130
Au	5.8949555649	8.3494952425	6.9824705008
Au	7.0592263836	9.7658600525	4.6911465272
Au	6.4588988185	6.5689948581	4.6722511394
Au	4.5096641775	8.5480064635	4.6457902380

Cartesian Coordinates of Au_{20}-MgO-reg

Au	12.3734027783	11.5257531985	4.6874188567
Au	7.8470072026	6.1772969422	7.0754816392
Au	9.2178775848	7.8236365575	11.3172113048
Au	10.4060380981	9.1483912655	9.1997956439
Au	11.5269792237	10.2568375893	6.9840123346
Au	9.7351162778	6.1496385694	9.2034503117
Au	11.1444507650	7.4206166090	7.0825207333
Au	12.0759143840	8.5966858940	4.6722027038
Au	10.2737955752	4.6075897645	7.0593681504
Au	11.3226316534	5.9425881827	4.7216707463
Au	10.7690490315	3.1560489255	4.7461022280
Au	7.4820091423	8.0907345550	9.1669729643
Au	8.6067959954	9.6447355279	7.0833390114
Au	9.7431891583	10.5591142279	4.6791566744
Au	9.0918056825	7.7456880128	4.8610407055
Au	8.4817405058	4.7332756869	4.7223590879
Au	5.9523834298	8.3651932344	6.9525313208
Au	7.0693029287	9.8995554550	4.7420597005
Au	6.4378938170	6.5158520162	4.6895828205
Au	4.5702034916	8.5722156367	4.6188215925
Mg	8.42200000	6.31650000	2.10550000
Mg	8.42200000	2.10550000	2.10550000
Mg	8.42200000	10.52750000	2.10550000
Mg	6.31650000	8.42200000	2.10550000
Mg	6.31650000	4.21100000	2.10550000
Mg	6.31650000	12.63300000	2.10550000
Mg	4.21100000	6.31650000	2.10550000

Mg	4.21100000	2.10550000	2.10550000
Mg	4.21100000	10.52750000	2.10550000
Mg	2.10550000	8.42200000	2.10550000
Mg	2.10550000	4.21100000	2.10550000
Mg	2.10550000	12.63300000	2.10550000
Mg	12.63300000	6.31650000	2.10550000
Mg	12.63300000	2.10550000	2.10550000
Mg	12.63300000	10.52750000	2.10550000
Mg	10.52750000	8.42200000	2.10550000
Mg	10.52750000	4.21100000	2.10550000
Mg	10.52750000	12.63300000	2.10550000
Mg	8.42200000	8.42200000	0.00000000
Mg	8.42200000	4.21100000	0.00000000
Mg	8.42200000	12.63300000	0.00000000
Mg	6.31650000	6.31650000	0.00000000
Mg	6.31650000	2.10550000	0.00000000
Mg	6.31650000	10.52750000	0.00000000
Mg	4.21100000	8.42200000	0.00000000
Mg	4.21100000	4.21100000	0.00000000
Mg	4.21100000	12.63300000	0.00000000
Mg	2.10550000	6.31650000	0.00000000
Mg	2.10550000	2.10550000	0.00000000
Mg	2.10550000	10.52750000	0.00000000
Mg	12.63300000	8.42200000	0.00000000
Mg	12.63300000	4.21100000	0.00000000
Mg	12.63300000	12.63300000	0.00000000
Mg	10.52750000	6.31650000	0.00000000
Mg	10.52750000	2.10550000	0.00000000

Mg	10.52750000	10.52750000	0.00000000
O	8.42200000	8.42200000	2.10550000
O	8.42200000	4.21100000	2.10550000
O	8.42200000	12.63300000	2.10550000
O	6.31650000	6.31650000	2.10550000
O	6.31650000	2.10550000	2.10550000
O	6.31650000	10.52750000	2.10550000
O	4.21100000	8.42200000	2.10550000
O	4.21100000	4.21100000	2.10550000
O	4.21100000	12.63300000	2.10550000
O	2.10550000	6.31650000	2.10550000
O	2.10550000	2.10550000	2.10550000
O	2.10550000	10.52750000	2.10550000
O	12.63300000	8.42200000	2.10550000
O	12.63300000	4.21100000	2.10550000
O	12.63300000	12.63300000	2.10550000
O	10.52750000	6.31650000	2.10550000
O	10.52750000	2.10550000	2.10550000
O	10.52750000	10.52750000	2.10550000
O	8.42200000	6.31650000	0.00000000
O	8.42200000	2.10550000	0.00000000
O	8.42200000	10.52750000	0.00000000
O	6.31650000	8.42200000	0.00000000
O	6.31650000	4.21100000	0.00000000
O	6.31650000	12.63300000	0.00000000
O	4.21100000	6.31650000	0.00000000
O	4.21100000	2.10550000	0.00000000
O	4.21100000	10.52750000	0.00000000

O	2.10550000	8.42200000	0.00000000
O	2.10550000	4.21100000	0.00000000
O	2.10550000	12.63300000	0.00000000
O	12.63300000	6.31650000	0.00000000
O	12.63300000	2.10550000	0.00000000
O	12.63300000	10.52750000	0.00000000
O	10.52750000	8.42200000	0.00000000
O	10.52750000	4.21100000	0.00000000
O	10.52750000	12.63300000	0.00000000

Cartesian Coordinates of Au_{20}-MgO-Ovac

Au	12.1983964867	11.5669418615	4.7326687300
Au	7.7702356225	6.2700941182	7.0491017794
Au	9.0932662632	7.8044988202	11.3726123783
Au	10.2247852973	9.2277178484	9.2922830959
Au	11.3191267162	10.4025073413	7.0989372861
Au	9.5800945187	6.0945369345	9.2632368372
Au	10.6033147101	7.5489476199	7.0436786517
Au	11.6263504638	8.7667444877	4.7501319967
Au	10.1506377811	4.6444300179	7.0462775591
Au	10.6680927081	6.2188484669	4.6630734384
Au	10.6201927058	3.2012615396	4.7409229862
Au	7.3746703391	8.0888264409	9.2069135052
Au	8.4631813187	9.6082239559	7.0786600253
Au	9.4636625439	10.9636209395	4.8709762797
Au	8.4787738131	8.3458841699	3.7773493780
Au	8.2334194887	4.6392960145	4.7879960302
Au	5.8549315679	8.4320837892	6.9759383852
Au	6.7873337475	10.3346994922	4.9427845606
Au	6.3230549154	6.5366768951	4.7437002339
Au	4.5282215315	8.6684027697	4.6211152639
Mg	8.42200000	6.31650000	2.10550000
Mg	8.42200000	2.10550000	2.10550000
Mg	8.42200000	10.52750000	2.10550000
Mg	6.31650000	8.42200000	2.10550000
Mg	6.31650000	4.21100000	2.10550000
Mg	6.31650000	12.63300000	2.10550000
Mg	4.21100000	6.31650000	2.10550000

Mg	4.21100000	2.10550000	2.10550000
Mg	4.21100000	10.52750000	2.10550000
Mg	2.10550000	8.42200000	2.10550000
Mg	2.10550000	4.21100000	2.10550000
Mg	2.10550000	12.63300000	2.10550000
Mg	12.63300000	6.31650000	2.10550000
Mg	12.63300000	2.10550000	2.10550000
Mg	12.63300000	10.52750000	2.10550000
Mg	10.52750000	8.42200000	2.10550000
Mg	10.52750000	4.21100000	2.10550000
Mg	10.52750000	12.63300000	2.10550000
Mg	8.42200000	8.42200000	0.00000000
Mg	8.42200000	4.21100000	0.00000000
Mg	8.42200000	12.63300000	0.00000000
Mg	6.31650000	6.31650000	0.00000000
Mg	6.31650000	2.10550000	0.00000000
Mg	6.31650000	10.52750000	0.00000000
Mg	4.21100000	8.42200000	0.00000000
Mg	4.21100000	4.21100000	0.00000000
Mg	4.21100000	12.63300000	0.00000000
Mg	2.10550000	6.31650000	0.00000000
Mg	2.10550000	2.10550000	0.00000000
Mg	2.10550000	10.52750000	0.00000000
Mg	12.63300000	8.42200000	0.00000000
Mg	12.63300000	4.21100000	0.00000000
Mg	12.63300000	12.63300000	0.00000000
Mg	10.52750000	6.31650000	0.00000000
Mg	10.52750000	2.10550000	0.00000000

Mg	10.52750000	10.52750000	0.00000000
O	8.42200000	4.21100000	2.10550000
O	8.42200000	12.63300000	2.10550000
O	6.31650000	6.31650000	2.10550000
O	6.31650000	2.10550000	2.10550000
O	6.31650000	10.52750000	2.10550000
O	4.21100000	8.42200000	2.10550000
O	4.21100000	4.21100000	2.10550000
O	4.21100000	12.63300000	2.10550000
O	2.10550000	6.31650000	2.10550000
O	2.10550000	2.10550000	2.10550000
O	2.10550000	10.52750000	2.10550000
O	12.63300000	8.42200000	2.10550000
O	12.63300000	4.21100000	2.10550000
O	12.63300000	12.63300000	2.10550000
O	10.52750000	6.31650000	2.10550000
O	10.52750000	2.10550000	2.10550000
O	10.52750000	10.52750000	2.10550000
O	8.42200000	6.31650000	0.00000000
O	8.42200000	2.10550000	0.00000000
O	8.42200000	10.52750000	0.00000000
O	6.31650000	8.42200000	0.00000000
O	6.31650000	4.21100000	0.00000000
O	6.31650000	12.63300000	0.00000000
O	4.21100000	6.31650000	0.00000000
O	4.21100000	2.10550000	0.00000000
O	4.21100000	10.52750000	0.00000000
O	2.10550000	8.42200000	0.00000000

O	2.10550000	4.21100000	0.00000000
O	2.10550000	12.63300000	0.00000000
O	12.63300000	6.31650000	0.00000000
O	12.63300000	2.10550000	0.00000000
O	12.63300000	10.52750000	0.00000000
O	10.52750000	8.42200000	0.00000000
O	10.52750000	4.21100000	0.00000000
O	10.52750000	12.63300000	0.00000000
